# Genomic prediction of trait segregation in a progeny population: a case study of Japanese pear (*Pyrus pyrifolia*)

**DOI:** 10.1186/1471-2156-14-81

**Published:** 2013-09-12

**Authors:** Hiroyoshi Iwata, Takeshi Hayashi, Shingo Terakami, Norio Takada, Toshihiro Saito, Toshiya Yamamoto

**Affiliations:** 1 Graduate School of Agricultural and Life Sciences, The University of Tokyo, 1-1-1 Yayoi, Bunkyo, 113-8657, Tokyo, Japan; 2 NARO Agricultural Research Center, 3-1-1 Kannondai, Ibaraki, 305-8666, Tsukuba, Japan; 3 NARO Institute of Fruit Tree Science, 2-1 Fujimoto, Ibaraki, 305-8605, Tsukuba, Japan

**Keywords:** Genomic selection, Selection of a parental combination, Segregation simulation, Bayesian modeling, Markov Chain Monte Carlo (MCMC), Genome-wide markers, Ordinal categorical scores

## Abstract

**Background:**

In cross breeding, it is important to choose a good parental combination that has high probability of generating offspring with desired characteristics. This study examines a method for predicting the segregation of target traits in a progeny population based on genome-wide markers and phenotype data of parental cultivars.

**Results:**

The proposed method combines segregation simulation and Bayesian modeling for genomic selection. Marker segregation in a progeny population was simulated based on parental genotypes. Posterior marker effects sampled via Markov Chain Monte Carlo were used to predict the segregation pattern of target traits. The posterior distribution of the proportion of progenies that fulfill selection criteria was calculated and used for determining a promising cross and the necessary size of the progeny population. We applied the proposed method to Japanese pear (*Pyrus pyrifolia* Nakai) data to demonstrate the method and to show how it works in the selection of a promising cross. Verification using an actual breeding population suggests that the segregation of target traits can be predicted with reasonable accuracy, especially in a highly heritable trait. The uncertainty in predictions was reflected on the posterior distribution of the proportion of progenies that fulfill selection criteria. A simulation study based on the real marker data of Japanese pear cultivars also suggests the potential of the method.

**Conclusions:**

The proposed method is useful to provide objective and quantitative criteria for choosing a parental combination and the breeding population size.

## Background

Both in self-pollinating and out-crossing plants, the selection of a good parental combination is an important breeding step that determines the degree of success achieved by the program because genetic variability in a progeny population is a key resource for obtaining superior genotypes [1]. Although the selection of a parental combination is an important decision in breeding, breeders usually find difficulty in identifying the best combination because they have insufficient information about the potential of the cross. A method enabling the prediction of (i) promising parental combinations, (ii) population size required, and (iii) genetic gain achievable with selection can help breeders to select a good parental combination in a reasonable manner. When breeding operations such as the establishment of a segregating population and the field evaluation of the population require larger amounts of time and cost, systematic planning for selecting crosses becomes even more important. For instance, in the breeding of a perennial tree such as Japanese pear (*Pyrus pyrifolia* Nakai), the field evaluation of a segregating population requires a long time and huge area because of its prolonged juvenile phase and large body [2,3]. Therefore, parental combinations should be determined as closely to ideally as possible.

Recently, a novel technology called genomic selection [4] has garnered increasing attention for use in plant and animal breeding. In genomic selection, genome-wide marker polymorphisms are used for predicting the genetic potentials of lines and individuals that have not been evaluated in field tests [5-7]. More specifically, based on the association between genome-wide marker polymorphisms **x**_*i*_ and phenotypic values *y*_*i*_ (*i* = 1, 2, …, *n*) of breeding lines, we can build a function relating the genetic value *g*_*i*_ to genome-marker polymorphisms **x**_*i*_, i.e. *g*_*i*_ = *f*(**x**_*i*_), under the assumption that *y*_*i*_ denotes the combined effect of *g*_*i*_ and environmental deviation *e*_*i*_, i.e. *y*_*i*_ = *g*_*i*_ + *e*_*i*_. Using the function *f*(**·**), it is possible to predict the genetic values *g*_*j*_ of an individual/line *j* based on its genome-wide marker polymorphisms **x**_*j*_, even when the individual/line *j* has no phenotypic record. Because genomic selection requires no phenotypic record of individuals and lines under the selection, it enables us (i) to select individuals and lines in an early developmental stage, and (ii) to select individual and lines without the influence of the environment. These characteristics make genomic selection more beneficial than conventional phenotypic selection from various perspectives [5-7], especially in perennial tree breeding [8-15].

We can estimate the genetic value of individuals with the arbitrary genotypes of genome-wide markers. Therefore, a genomic selection prediction model will enable us not only to predict the potential of the lines and individuals under selection, but also to predict the potentials of “virtual” lines and individuals if the lines and individuals have genome-wide marker genotypes. Using this mechanism, we can predict the segregation of target traits in a progeny population based on simulated segregation of genome-wide markers, which suggests that genomic selection is useful not only for selecting promising individual and lines but also for selecting promising cross combinations based on the predicted segregation of target traits. This idea seems useful for designing breeding programs in a systematic manner, but relatively little [16,17] are known to date.

In this paper, we propose a novel method for predicting the segregation of target traits and for selecting promising parental combinations based on the prediction. For the proposed method, we used the genotypes of genome-wide markers and the phenotypes of target traits in a population of parental cultivars for training a prediction model. A key point of the method is that we combined the segregation simulation of marker genotypes and Bayesian regression via Markov chain Monte Carlo (MCMC) sampling: the segregations of genome-wide markers in a progeny population were simulated based on phased haplotypes estimated for parental cultivars and the positions of markers on the linkage map. The segregations of target traits were then predicted by plugging the MCMC samples of marker effects into regression equations based on simulated marker segregation data. The method enables us to calculate the posterior distribution of the proportion of progenies that fulfill specified selection criteria. We can use the posterior proportion to determine the promising crosses and the necessary size of a progeny population.

In this study, we applied the proposed method to data collected from parental cultivars used in Japanese pear breeding programs. Japanese pear, an important fruit tree, has the third greatest share of fruit production (258,700 t in the 2010 fiscal year) in Japan [18]. Although two cultivars, ‘Kosui’ and ‘Hosui’, account for more than 60% of the share of Japanese pear production, a new cultivar compensating for shortcomings of these two cultivars is eagerly anticipated. To accelerate the genetic improvement of Japanese pear, genetic markers and their linkage maps have been developed and used for the genetic dissection of complex traits [19-25]. The possibilities indicated by results of genome-wide association studies and genomic prediction have also been investigated in Japanese pear [15]. For the present study, we used Japanese pear data to demonstrate our proposed method and to explain how it works in the selection of promising crosses. As a proof-of-concept study, we compared real and predicted segregation of harvest time and fruit weight in an actual breeding population to validate the potential prediction ability of the method. We also conducted a simulation study based on actual marker data of the Japanese pear cultivars. In the study, we simulated the phenotypes of the cultivars based on quantitative trait loci (QTL) placed at randomly selected markers, and applied the proposed method to predict the proportion of progenies that fulfill a selection criterion. The prediction accuracy was evaluated by comparing the true and predicted proportion.

## Results

**Figure 1:**
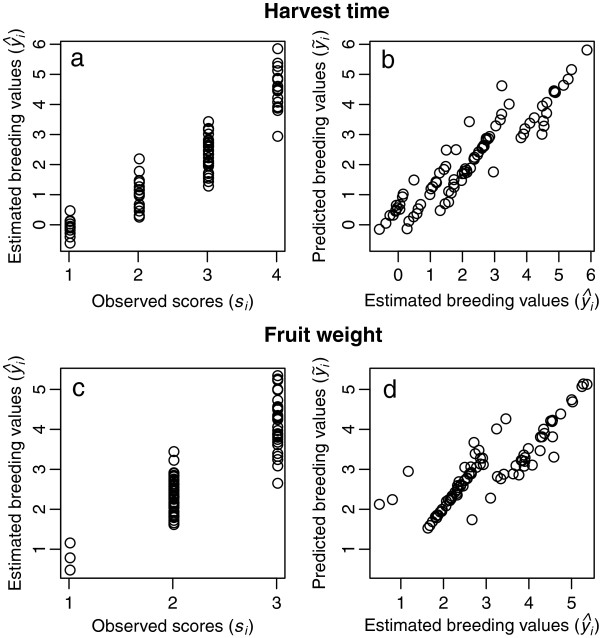
**Estimated and predicted breeding values and observed scores in harvest time (a and b) and fruit weight (c and d) in the population of 84 cultivars.** Relations between estimated breeding values and observed scores **(a and c)** between predicted and estimated breeding values **(b and d)**.

Using trait phenotype and marker genotype data of the 84 Japanese pear cultivars, we developed genomic prediction models for harvest time and fruit weight. The phenotypic data of both traits were recorded as ordinal categorical scores. We built a prediction model that regressed a latent continuous variable on genome-wide marker polymorphisms [15,26] for each trait, and estimated the latent continuous variable as the breeding values of the 84 cultivars for the trait. Correlation coefficients between the estimated breeding values and the observed categorical scores were 0.93 and 0.88, respectively, for harvest time and fruit weight, indicating that the regression models fitted the observed data well (Figures 1a, c). Using these regression models, we estimated the prediction accuracy by calculating the predicted breeding values and ordinal categorical scores via leave-one-out cross-validation for the two traits. Correlation coefficients obtained between the predicted and estimated breeding values were 0.94 and 0.87 for harvest time and fruit weight, respectively, indicating that both traits can be predicted accurately and that the prediction is more accurate for harvest time than for fruit weight (Figures 1b, d). The degrees of coincidence between the predicted and observed ordinal categorical scores were, respectively, 0.54 and 0.72 for harvest time and fruit weight, respectively (Table 1). In fruit weight, the lowest score class could not be predicted accurately (Figure 1d and Table 1). Low accuracy might be caused by large environmental error in the class or the small number of cultivars included in the class. For consideration of the dominance effect as well as additive effects, we also applied a genotype effect model [27] in the prediction. The prediction accuracy of the model, however, was lower than that of the additive effect model (*r* = 0.92 and 0.78 in terms of harvest time and fruit weight, respectively).

**Table 1:** Coincidence between predicted and observed scores of ordinal categorical data in harvest time and fruit weight

**Observed scores**	**Predicted scores**			
Harvest time	1	2	3	4
1	4	4	0	0
2	7	2	3	0
3	2	11	29	9
4	0	0	3	10
Fruit weight	1	2	3	
1	0	0	0	
2	3	42	15	
3	0	5	17	

**Figure 2:**
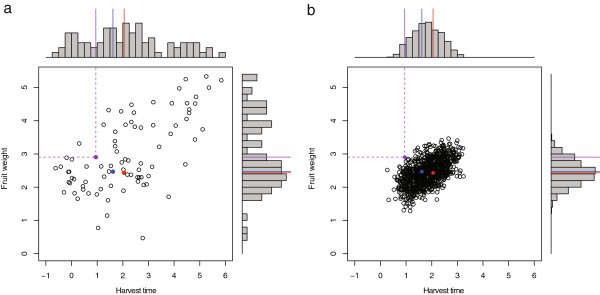
**Genomic breeding values of parental cultivars and predicted segregation in an F**_**1**_** progeny population. (a)** Estimated genomic breeding values of 84 parental cultivars in harvest time and fruit weight. **(b)** Predicted segregation in an F_1_ progeny population derived from the cross ‘Kikusui’ × ‘Wase-Kouzou’. ‘Kikusui’ (red dots in **a** and **b**) ‘Wase-Kouzou’ (blue dots) are parents of the most major cultivar ‘Kosui’ (purple dots).

Based on the phased marker genotypes of the 84 Japanese pear cultivars, we simulated genome-wide marker segregation in a segregating population for each of the possible combinations of the 84 cultivars. Using the genomic prediction models, we predicted the segregation of breeding values in harvest time and fruit weight in each of segregating populations based on the simulated marker segregation of the population. Here, as an example, we present the prediction result of an F_1_ population from the cross between two cultivars ‘Kikusui’ and ‘Wase-Kouzou’, which are the parents of the most major cultivar ‘Kosui’. Figure 2a shows the estimated genomic breeding values of 84 cultivars, which include ‘Kikusui’, ‘Wase-Kouzou’, and ‘Kosui’, in harvest time and fruit weight. Among the 84 cultivars, ‘Kikusui’ and ‘Wase-Kouzou’ have medium harvest time, whereas ‘Kosui’ has early harvest time. All three cultivars have medium fruit weight (Figure 2a). Figure 2b shows the predicted segregation pattern of harvest time and fruit weight in an F_1_ progeny population derived from the cross ‘Kikusui’ × ‘Wase-Kouzou’. The predicted segregation suggests that the cross has transgressive segregation, where some progenies have more extreme phenotypes than either parent, in both harvest time and fruit weight and that the early harvest time of ‘Kosui’ can be explained well by the transgressive segregation of this cross. Under the predicted segregation of both traits, the posterior proportion of progenies that have earlier harvest time and larger fruit weight than ‘Kosui’ was less than 0.1% (i.e., none of 1,000 progenies fulfilled the criteria) from the cross ‘Kikusui’ × ‘Wase-Kouzou’, suggesting that ‘Kosui’ is a superior progeny derived from the cross. Both Figures 2a, b show a trade-off relationship between harvest time and fruit weight, i.e., earlier harvest time is associated with smaller fruit weight in both the population of parental cultivars and in a segregating population. Genetic correlation coefficients between two traits were estimated as 0.622 and 0.559, respectively, for the parental population (Figure 2a) and the progeny population (Figure 2b), which makes it somewhat difficult to obtain progenies with earlier harvest time and greater fruit weight than ‘Kosui’.

**Figure 3:**
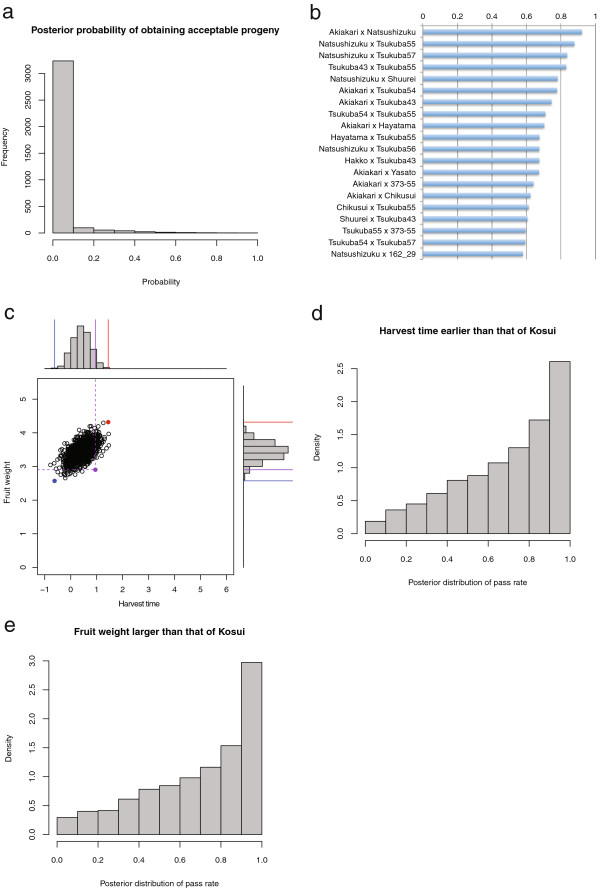
**Predicted segregation of target traits and posterior probability of obtaining progenies fulfilling selection criteria. (a)** Posterior probability of obtaining acceptable progenies for all combinations of 84 cultivars. **(b)** Top 20 crosses with a high probability of obtaining acceptable progenies. **(c)** Predicted segregation of harvest time and fruit weight in a population derive from the most promising cross ‘Akiakari’ × ‘Natsushizuku’. **(d)** Posterior probability of obtaining progenies fulfilling the harvest time criterion (i.e., earlier than ‘Kosui’) under the most promising cross. **(e)** Posterior probability of obtaining progenies fulfilling the fruit weight criterion (i.e., larger than ‘Kosui’) under the most promising cross.

Using the proposed method, we calculated the proportion of progenies that have earlier harvest time and larger fruit weight than ‘Kosui’ for all possible crosses among the 84 cultivars (3,486 combinations). For most crosses (3,236, 93%), the proportion was less than 10% (Figure 3a). Of these, 2,403 crosses had less than 0.1% of the proportion. Figure 3b shows the top 20 crosses with a high proportion of progenies that fulfill the criteria for harvest time and fruit weight. These crosses had more than 50% of the proportion, suggesting that not every cross combination results in a low proportion of progenies fulfilling the criteria. Among the 3,486 crosses, the cross ‘Akiakari’ × ‘Natsushizuku’ showed the highest probability (0.921). Of the top 20 crosses, the cultivars ‘Akiakari’ and ‘Natsushizuku’ appeared 7 and 6 times, respectively, suggesting that these two cultivars are key parents to breed early and large fruit cultivars. Figure 3c shows the predicted segregation pattern of harvest time and fruit weight in an F_1_ progeny population derived from the cross ‘Akiakari’ × ‘Natsushizuku’. The predicted segregation pattern indicated that this cross combination had a low frequency of transgressive segregation in both harvest time and fruit weight, although most progenies fulfill the criteria. To estimate the degree of uncertainty of the prediction, we calculated the posterior distribution of the proportion of progenies that fulfill the criteria (Figures 3d, e). In both harvest time and fruit weight, the mean posterior proportion was high (0.690 in both traits). Moreover, its distribution was biased to one, suggesting that progenies have high probability of fulfilling the criteria.

**Figure 4:**
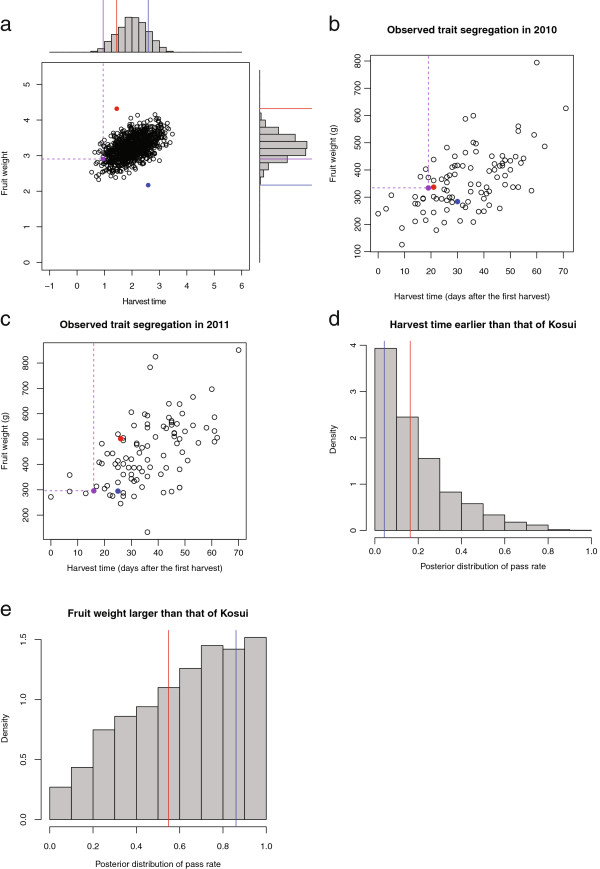
**Predicted and observed segregation in a real breeding population and estimated posterior probability of obtaining progenies fulfilling selection criteria. (a)** Predicted segregation of harvest time and fruit weight in a population derived from the cross ‘Akiakari’ × ‘Taihaku’. **(b and c)** Segregation of harvest time and fruit weight observed in 2010 and 2011. Data were measured on continuous scales (i.e., they were not values of latent variables estimated from ordinal categorical data). **(d)** Posterior probability of obtaining progenies fulfilling the harvest time criterion (i.e., earlier than ‘Kosui’) under the cross ‘Akiakari’ × ‘Taihaku’. Vertical lines represent realized proportions observed in 2010 (red line) and 2011 (blue line). **(e)** Posterior probability of obtaining progenies fulfilling the fruit weight criterion (i.e., larger than ‘Kosui’) under the cross ‘Akiakari’ × ‘Taihaku’. Vertical lines represent realized proportions observed in 2010 (red line) and 2011 (blue line).

To verify the potential of the proposed method, we applied the method to the prediction of segregation pattern of harvest time and fruit weight in a real breeding population and compared it to the observed segregation pattern of both traits. The breeding population was an F_1_ progeny population derived from the cross between ‘Akiakari’ × ‘Taihaku’. Figure 4a shows the predicted segregation pattern, whereas Figures 4b, c respectively show the segregation patterns observed in 2010 and 2011. From the prediction of segregation pattern, transgressive segregation can be expected in harvest time but not in fruit weight. Although the estimated breeding values of two parents (red and blue dots in Figures 4a, b, c) were not well accorded with their observations (i.e. the parents have extreme values in the estimations but not in the observations) in fruit weight, the predicted segregation pattern agreed well with the segregation patterns observed in 2010 and 2011. In the predicted pattern, the proportion of progenies that fulfilled the criteria was 0.6% (i.e., 6 out of 1,000 progenies fulfilled the criteria). In the segregation patterns observed in 2010 and 2011, actually, the respective proportions of progenies fulfilling the criteria were 3.3% and 1.1%. Although the observed values were slightly larger than the predicted values, both values are mutually consistent at a practical level. To estimate the degree of uncertainty of the prediction, we calculated the posterior distribution of the proportion of progenies that fulfill the criteria (Figures 4d, e). The observed proportions of progenies that fulfill the criteria seem to follow the posterior distribution both in harvest time and fruit weight: in harvest time, the posterior distribution is biased toward zero. The observed proportions were small (0.163) in 2010 and close to zero (0.043) in 2011. In fruit weight, the posterior distribution was not so sharp. The observed proportions have greatly differing values between the two years (0.548 and 0.860 in 2010 and 2011, respectively). These results suggest that the shape of the posterior distribution is a good indicator reflecting the degree of uncertainty of the predicted segregation.

**Figure 5:**
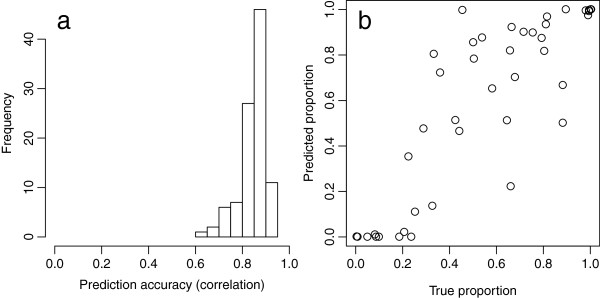
**Summary of a simulation study based on the marker genotype data of 84 Japanese pear cultivars. (a)** Correlation between the true (i.e., simulated) and predicted proportions of progenies fulfilling the selection criterion in 100 simulations. **(b)** True and predicted proportions of progenies fulfilling the selection criterion in the simulation that showed average accuracy (i.e., the simulation with accuracy was close to the average of 100 simulations).

To verify the potential of the proposed method, we also conducted a simulation study based on the marker genotype data of the 84 Japanese pear cultivars. In the study, we simulated the genotypes and phenotypes of the 84 Japanese pear cultivars based on QTL placed at randomly selected markers, and applied the proposed method to predict the proportion of progenies that fulfill a selection criterion. Results show that correlation between the true and predicted proportion was high (0.84 in average; Figure 5a). Figure 5b is the relationship between the true and predicted proportion in the simulation that showed average accuracy.

## Discussion

Using the proposed method, we can predict the segregation pattern of target traits in a segregating population based on genomic prediction models, genome-wide marker genotype data, and linkage map data. Based on the prediction of a segregation pattern, we can calculate the probability of obtaining genotypes with characteristics required for new cultivars. The prediction always includes uncertainty because of the limited number of samples in training data and the environmental variations masking true genotypic values of training samples. The degree of uncertainty differs among traits, depending on the heritability and the genetic system of the traits. The degree of uncertainty is also different among parental combinations, depending on the QTL and marker genotypes of parental cultivars. Using the MCMC sampling algorithm, we can estimate the degree of uncertainty by calculating a posterior distribution for the proportion of progenies with desired characteristics. This information is expected to be useful for breeders to choose a good parental combination that has high probability of generating offspring with desired characteristics.

Various statistical methods have been proposed for selecting cross combinations [1]. Most, however, are methods for predicting the average potential of a progeny population (e.g., the potential of F_1_ hybrid lines generated from two inbred lines), and are not methods for predicting the segregation pattern in a progeny population. The range of segregation of target traits differs among cross combinations. Therefore, the average potential alone cannot be sufficient information for selecting parental combinations. The method proposed in this study, in contrast, enables us to predict the segregation pattern in a progeny population, and thereby provide more detailed information about the cross combinations. For example, with the segregation pattern prediction, breeders can select parental combinations expecting transgressive segregation in a segregated population. Moreover, breeders can postulate the necessary size of a segregated population to obtain superior progenies. The expected genetic gain can also be calculated from the segregation pattern prediction. Quantitative and objective information about crosses will provide breeders a reasonable means to select parental combinations. In fruit tree breeding, techniques of asexual propagation are commonly used. Therefore, a good F_1_ progeny becomes a cultivar directly using the asexual propagation. It is important to predict the range of segregation in an F_1_ progeny population rather than the average genetic potential of progenies in the population.

For this study, we applied the proposed method to the segregation of harvest time and fruit weight in an actual breeding population derived from the cross ‘Akiakari’ × ‘Taihaku’. Segregation patterns observed in 2010 and 2011 agreed well with the predicted segregation pattern, suggesting the potential of the proposed method for predicting the segregation of target traits in a progeny population. The degree of uncertainty of the predicted segregation was calculated as a posterior distribution of the proportion of progenies that fulfill the criteria. It was compared with the observed proportions. Consequently, the observed proportion of progenies that fulfill the criteria seems to follow the posterior distribution. In the breeding population, the posterior distribution of fruit weight showed a broader peak than that of harvest time, suggesting that the uncertainty of the predicted proportion was larger in fruit weight than in harvest time possibly because fruit weight has lower heritability than at harvest time. As this example showed, the degree of uncertainty will differ depending on traits and cross combinations. Therefore, it is important to provide information about the uncertainty of the prediction for each trait and each cross combination. Especially when the number of markers and the number of samples used for building a prediction model, the uncertainty of the prediction can be large and therefore should be considered when breeders select cross combinations. Because the proposed method was validated based on one breeding population, additional studies will be necessary to evaluate the potential of the method in other plant species as well as Japanese pear.

For this study, we used BayesA for building a prediction model and BEAGLE for estimating phased genotypes of parental cultivars and lines. Moreover, several alternative methods for conducting the equivalent calculation exist. For example, we used BayesB, which is a model assuming that most markers have no effects on genetic values, as well as BayesA in our previous study [15]. In our previous study, BayesA performed better than BayesB in most traits, partly because of the low density of markers used in the prediction. BayesB is known to perform better when the linkage disequilibrium between QTL and markers is stronger [28]. Therefore, the advantage of BayesB over BayesA might appear when the number of markers is sufficiently large to ensure strong linkage disequilibrium between QTL and markers. When linkage disequilibrium between QTL and markers is weak, random regression best linear unbiased prediction (RR-BLUP) is expected to yield better than either BayesA and BayesB [29-31]. Applying RR-BLUP to the present dataset revealed that RR-BLUP had lower accuracy than BayesA (data not shown). The result indicated that genotypic variations in the traits analyzed in this study could be explained by linkage equilibrium between markers and QTL as well as kinship relationships among cultivars. In fact, two markers showed significant association with variations in harvest time in a genome-wide association study using 76 Japanese pear cultivars [15], suggesting linkage disequilibrium between the significant markers and QTL.

Non-additive effects, i.e., dominance and epistasis, are also important for the selection of a good parental combination. Because of their importance, Lü et al. [16] proposed a method for the prediction of elite cross combinations by considering epistasis. In fruit tree breeding, breeders can exploit all genetic effects, i.e., additive and non-additive, as they are expressed in the phenotypes of individuals [3], because the superior individuals can be propagated by asexual means. In this study, we applied the genotype effect model [27], which can include both additive and dominance effects as “genotype effects” of markers. The prediction accuracy of the model was equivalent to that of the additive allelic model, suggesting that dominance effects were small in traits analyzed in this study. It is, however, also possible that the sample size is not sufficiently large to estimate numerous genotype effects for multi-allelic markers accurately. When the samples are few, it is difficult to model dominance and epistasis effects explicitly because the number of possible models is too large. In that case, nonlinear kernel methods might be a good alternative of models involving the nonlinear effects. For instance, reproducing kernel Hilbert space regression [32,33] is a promising nonlinear kernel regression method [34]. To estimate the phased genotypes of parental cultivars, we used BEAGLE in this study because most markers were multi-allelic. When markers are bi-allelic, other algorithms, such as fastPHASE [35] and MaCH [36], can be good alternatives to BEAGLE. Methods and algorithms used in the proposed method are currently advancing at a fast pace. Therefore, the advent of novel methods and algorithms will further improve the accuracy of the proposed method.

In this study, we used the Bayesian latent variable regression to estimate genetic effects of multiple markers. The method is useful for the analysis of data collected in breeding programs because field-testing data are often collected as ordinal categorical or binary data to save labor for measuring traits. The Bayesian method has been applied to the QTL analysis [37,38] and genome-wide association studies [15,26,39] of binary and ordinal categorical traits. Iwata et al. [15] first applied the method to the genome-wide predictions of breeding values in ordinal traits in the context of genomic selection. In the present study, we extended the method further to the prediction of a segregation pattern in a progeny population. As described above, the Bayesian approach enables us to calculate the posterior distribution of the proportion of acceptable progenies via the MCMC sampling and to estimate the uncertainty of the prediction. When only the point estimate of the proportion is needed, fast algorithms proposed in earlier reports [37-39] will be useful especially when the markers are numerous. Although the Bayesian method is useful to estimate genetic effects of markers in ordinal categorical data, it is noteworthy that ordinal categorical scoring can lose information that is necessary to estimate small genetic effects as described in an earlier report [26].

In this study, we used the dataset of the 84 Japanese pear cultivars to demonstrate the potential of the proposed method. The cultivars in the data are few and insufficient to build an accurate prediction model when heritability of a target trait is low. In this study, the analyzed target traits are thought to be highly heritable. Especially for harvest time, Iwata et al. [15] detected two significant markers via a genome-wide association study using data of 76 Japanese pear cultivars, and found that the markers collocated with a known gene and a QTL detected in a bi-parental F_1_ population. To predict the segregation pattern of a target trait accurately, however, numerous cultivars will be necessary.

Marker density in the 84 Japanese pear data is insufficient to ensure strong linkage disequilibrium between QTL and markers. Although the range of linkage disequilibrium in a Japanese pear population extends to about 10 cM [15], lower marker density induces more frequent recombination between markers and QTL, and worsen the prediction accuracy of the segregation pattern because the prediction model assigns genotypic effects to markers instead of unobservable QTL.

Recently, whole-genome genotyping using genome-wide markers has become inexpensive and operationally straightforward with high-throughput [40-43]. This trend will drive the actual use of the proposed method in the breeding of various crop plants. For this study, we used only the trait phenotype and marker genotype data of parental cultivars to build prediction models. The accuracy of the prediction models is expected to increase through the use of the trait phenotype and marker genotype data of progenies in segregating breeding populations.

## Conclusion

In this paper, we proposed a method for predicting the segregation pattern of target traits in a progeny population based on genomic prediction models. An empirical study using a real segregation population of Japanese pear suggested the usefulness of the proposed method. The certainty of predicted segregation pattern is thought to change depending on traits and cross combinations. By calculating the posterior distribution of the proportion of acceptable progenies, the degree of uncertainty of the prediction can be estimated. The method is expected to be useful to provide objective and quantitative criteria for determining parents for crossing and the size of a segregating population.

## Methods

### Japanese pear data

We used marker genotype and trait phenotype data of 84 Japanese pear cultivars. The 84 cultivars consisted of 37 modern elite cultivars, 20 old cultivars, 17 indigenous cultivars, and 10 breeding lines (Table 2). Of those, 76 had been analyzed in the context of genome-wide association study and the validation of prediction accuracy of genomic selection prediction models [15]. All plant materials were maintained and collected at the NARO Institute of Fruit Tree Science (NIFTS, Ibaraki, Japan).

**Table 2:** List of pear cultivars and breeding lines analyzed in the present study

**Name**	**Type**	**Release year**	**HT**^**†**^	**FW**^**‡**^	**Name**	**Type**	**Release year**	**HT**	**FW**
Akemizu	Modern	1997	1	2	Heiwa	Old		3	2
Akiakari	Modern	2003*	2	3	Inagi	Old		3	3
Akibae	Modern	1997	4	2	Kikusui	Old		3	2
Akizuki	Modern	2001*	3	3	Kimitsuka Wase	Old		1	2
Chikusui	Modern	1989*	1	2	Kogetsu	Old		3	-
Choju	Modern	1973	3	2	Kunitomi	Old		3	1
Hakko	Modern	1972*	2	3	Niitaka	Old		4	3
Hayatama	Modern	1968*	1	-	Sagami	Old		2	2
Hiratsuka-10	Modern		3	3	Seigyoku	Old		3	2
Hokushin	Modern	1997	3	2	Shinkou	Old	1941	4	2
Hosui	Modern	1972*	3	3	Shinseiki	Old		2	3
Hougetsu	Modern	1994*	4	3	Touno	Old		2	1
Kisui	Modern	1990	1	2	Yachiyo	Old		4	3
Kosui	Modern	1959*	2	2	Yanaga	Old		4	3
Kumoi	Modern	1955*	2	2	Amanogawa	Indigenous		4	3
Nangetsu	Modern	1997	3	3	Chojuro	Indigenous		3	2
Natsuhikari	Modern	1995	2	2	Doitsu	Indigenous		3	2
Natsushizuku	Modern	2005*	1	2	Ichihara Wase	Indigenous		2	2
Nikkori	Modern	1996	4	3	Imamuraaki	Indigenous		4	3
Oushuu	Modern	2003*	4	3	Ishii Wase	Indigenous		2	2
Shinsei	Modern	1984*	3	2	Kinchaku^§^	Indigenous		3	2
Shinsetsu	Modern	1949	4	3	Laiyangcili	Indigenous		4	3
Shinsui	Modern	1965*	2	2	Meigetsu	Indigenous		4	3
Shuugyoku	Modern	1988*	3	3	Mishirazu	Indigenous		3	3
Shuurei	Modern	2003*	3	3	Nijisseiki	Indigenous		3	2
Suisei	Modern	1955*	2	2	Okusankichi	Indigenous		4	3
Tama	Modern	1971	2	2	Seiryuu	Indigenous		4	3
Tsukuba43^§^	Modern		1	2	Shinchuu	Indigenous		2	1
Tsukuba52	Modern		3	2	Taihaku	Indigenous		3	2
Tsukuba53	Modern		3	2	Wase Kouzou	Indigenous		3	2
Tsukuba54^§^	Modern		1	2	Waseaka	Indigenous		4	2
Tsukuba55^§^	Modern		2	3	Yali	Indigenous		4	2
Tsukuba56^§^	Modern		3	3	42-6	Breeding line		2	2
Tsukuba57^§^	Modern		3	3	92-7	Breeding line		3	2
Tsukuba58^§^	Modern		4	3	162-29	Breeding line		3	3
Wakahikari	Modern	1992	1	2	266-27	Breeding line		3	2
Yasato	Modern	1990*	1	2	373-55^§^	Breeding line		1	2
Asahi	Old		3	2	48-96	Breeding line		3	2
Atago	Old		4	3	C2	Breeding line		3	3
Gion	Old		3	3	I-33	Breeding line		3	2
Hatsuaki	Old		3	2	O-9	Breeding line		1	2
Hattatsu	Old		3	2	Ri-14	Breeding line		2	2

^†^Harvest time.

^‡^Fruit weight.

^§^Cultivars and breeding lines that were not in the dataset analyzed in [15].

*Modern elite cultivars bred by the National Institute of Fruit Tree Science (NIFTS, Ibaraki, Japan).

Marker genotypic data consisted of genotypes of 333 markers of the 84 cultivars. In addition to the 155 simple sequence repeat (SSR) and three gene markers used for our previous report [15], 175 SSR markers were newly genotyped, including 59 pear EST-SSRs [44], 34 pear genomic SSRs of tetra-nucleotide and 38 pear genomic SSRs of penta-nucleotide motifs [44], 30 apple EST-SSRs [45], and 14 apple genomic SSRs [46]. The SSR-PCR amplification and genotyping by DNA sequencer were performed using methods described for an earlier study [15]. The mean and median of distances between adjacent markers (excluding markers located at the same positions) were, respectively, 4.0 and 2.7, respectively.

Traits analyzed in this study were harvest time and fruit weight, which are important traits in Japanese pear breeding. Both traits were recorded with ordinal categorical scores based on the plant genetic resource criteria [47]. Harvest time and fruit weight were recorded respectively with the four levels (i.e., 1–4) and three levels (i.e., 1–3) of scores. Two cultivars, “Kogetsu” and “Hayatama”, had missing data in fruit weight.

To verify the possibilities of the proposed method, we applied the method for predicting the segregation of harvest time and fruit weight in a real breeding population. The population consisted of 93 F_1_ progenies derived from the cross between ‘Akiakari’ and ‘Taihaku’. The F_1_ progenies, the two parental cultivars ‘Akiakari’ and ‘Taihaku’, and the most major cultivar ‘Kosui’ were cultivated in an experimental field in NIFTS. In 2010 and 2011, the harvest time and fruit weight of these progenies and cultivars were measured in continuous scales. Evaluations of the harvest time and fruit weight were conducted using 6-year-old trees for F_1_ progenies derived from the cross between ‘Akiakari’ and ‘Taihaku’. An original 26-year-old tree was used for ‘Akiakari’, and trees 18-years and 12-years after grafting were used, respectively, for ‘Taihaku’ and ‘Kosui’. They were grown and maintained using culture techniques incorporated into commercial pear production practices in Japan [48]. The trees were trained on horizontal trellises, pruned annually in winter, and treated for pests and diseases. Young fruits were thinned to 1 per 3 fruit clusters in mid-May, and harvested during early August to October according to a fruit ground color chart that shows the optimum color for harvesting Japanese pear [49]. The optimum fruit ground color was determined when the fruit skin color in the calyx end changes to the color of scale 4 of the color chart. Harvest time was determined as the central day between start and end for harvesting. Fruit weight was measured as the average weight of all harvested fruits.

### Outline of data analysis procedure

**Figure 6:**
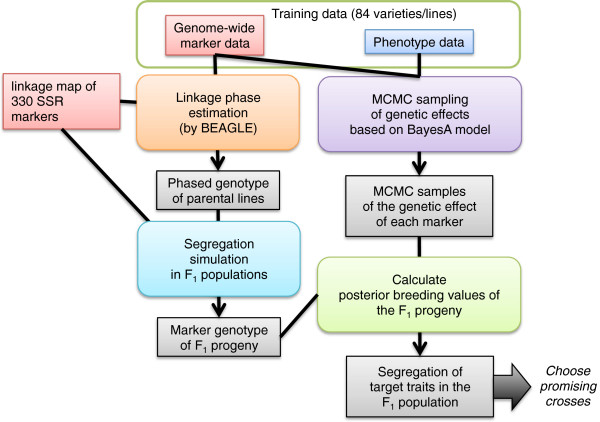
**Procedure for predicting the segregation patterns of target trait in progeny populations.** See text for details.

Figure 6 presents an overall picture of the proposed method. Data required for the method are genotype data of genome-wide markers, the phenotypic data of target traits, and the positions of the genome-wide markers on the linkage map. The marker and phenotypic data are assumed to be collected for all cultivars and lines that are candidate parents for crossing and their related cultivars and lines. Two main streams of data analysis exist: one for segregation simulation and the other for the estimation of effects of genome-wide markers.

### Segregation simulation

To simulate marker segregation in F_1_ populations, we first estimated the most likely linkage phases of 84 parental cultivars using BEAGLE [50]. Based on the most likely linkage phases and the linkage map positions of genome-wide markers, we simulated marker segregation in F_1_ populations for all combinations of the parental cultivars. We simulated 1,000 genotypes (i.e. progenies) of the genome-wide markers for each population. For the simulations, we inversely calculated a recombination rate between adjacent markers from distance between the markers on the linkage map using the Haldane’s map function [51] and simulated recombination events in each chromosome based on the calculated recombination rates for all marker intervals. We assumed no interference in genetic recombination.

### Estimation of effects of genome-wide markers

The statistical model used for this study is the same as that presented in our previous paper [15]. The model fundamentally had the form of the Bayesian shrinkage regression, which is also known as BayesA [4], but it incorporated a latent dependent variable for modeling an unrecorded continuous variable that underlies recorded ordinal scores. Here we explain the model briefly to make this paper self-explanatory. The regression model has the following form.

(1) $$y_{i}=\beta_{0}+\sum_{j=1}^{J}\sum_{l=1}^{L_{j}}\left(x_{\mathrm{ijl}}+x{'}_{\mathrm{ijl}}\right)\beta_{\mathrm{jl}}+e_{i}$$

In Equation (1), *y*_*i*_ is the latent continuous variable underlying the recorded ordinal scores *s*_*i*_ for the *i*th cultivar. *β*_0_ is the intercept. *L*_*j*_ represents the number of alleles of marker *j* (*j* = 1, 2,…, *J*), *x*_*ijl*_ and *x’*_*ijl*_ denotes the two alleles of marker *j* for cultivar/line *i*, and equals 1 if the allele is the *l*th allele (*l*=1, 2,…, *L*_*j*_) and 0 otherwise. *β*_*jl*_ denotes the genetic effect associated with the allele *l* of marker *j*, which is assumed to follow *N*(0, *σ*_*j*_^2^). *e*_*i*_ is the residual error, which is assumed to follow *N*(0, *σ*_*e*_^2^). The genetic variance of marker *j*, *σ*_*j*_^2^, and the error variance, *σ*_*e*_^2^, were assumed to follow scaled inverted chi-square distribution Inv-χ^2^(ν_*g*_, *S*_*g*_) and Inv-χ^2^ (ν_*e*_, *S*_*e*_). The latent continuous variable *y*_*i*_ is related to the ordinal score *s*_*i*_ as follows. When the phenotypic records were scored in *M* ordinal categories, the value of each *y*_*i*_ falls into one of *M* contiguous bins on the real line demarcated by the cut-points *K*_0_, *K*_1_,…, *K*_*M*_, and the observed values of *s*_*i*_ are determined using the following relation:

$$s_{i}=m\;\mathrm{if}\;K_{m}{}_{‒1}<y_{i}\leq K_{m}\left(m=1,2,\ldots ,M\right).$$

Because the cut-points are also unobservable, the values of *K*_*m*_ were also estimated, but the first, second, and last cut-points were fixed as *K*_0_ = −∞, *K*_1_ = 0, and *K*_*M*_ = ∞. A restriction on the cut points (i.e., *K*_1_ = 0) is necessary to ensure that all parameters are identifiable [52]. To assess the importance of dominance effects in traits analyzed in this study, we also built a model with the following form:

(2) $$y_{i}=\beta_{0}+\sum_{j=1}^{J}\sum_{g=1}^{G_{j}}\beta_{\mathrm{jg}}z_{i}{}_{\mathrm{jg}}+e_{i}$$

Therein, *z*_*ijg*_ denotes the genotype of marker *j* for cultivar/line *i*, and equals 1 if the genotype is the *g*th type (*g*=1, 2,…, *G*_*j*_) and 0 otherwise. *β*_*jg*_ denotes the genetic effect associated with the genotype *g* of marker *j*. Although the model does not include the dominance effects explicitly, the estimated genetic effect, *β*_*jg*_, can be regarded as the sum of additive and dominance effects of marker *j*[27]. Because of the multi-allelic nature of markers used in the Japanese pear data, we did not assign additive and dominance effects separately to all alleles and allelic combinations to avoid over-parameterization.

Parameters in the models were estimated via MCMC sampling. For each trait, MCMC cycles were repeated 50,000 times. The first 10,000 burn-in cycles were not used for parameter estimation. The sampling was conducted every ten cycles to reduce serial correlation. Consequently, the number of samples we retained was 4,000. The hyperparameters of the models were set as ν_*g*_ = 4, *S*_*g*_^*2*^ = 0.004, ν_*e*_ = −2, and *S*_*e*_^*2*^ = 0. This setting of hyperparameters was used in [15]. For the genotype effect model as Equation (2), we set *S*_*g*_^*2*^ = 0.012 to account for the fact that the genotype effect of a marker (the sum of dominance and additive effects of a marker) has larger variance than the additive effect of a marker allele.

Using the Bayesian regression described above, we calculated the estimated breeding values (*ŷ*_*i*_), which is the expectation of latent continuous variable *y*_*i*_, using whole data. To the accuracy of genomic prediction (i.e., prediction based on the regression), we calculated predicted breeding values (*ỹ*_*i*_), which is the prediction of the variable *y*_*i*_, via leave-one-out cross-validation. The accuracy of genomic prediction was measured as Pearson’s product moment correlation coefficients between *ỹ*_*i*_ and *ŷ*_*i*_. We also calculated the predicted ordinal scores (${\tilde{s}}_{i}$) by assigning the *i*th cultivar to the most probable score category based on the MCMC samples of *s*_*i*_ (i.e., the category most frequently sampled in the MCMC sampling). The prediction accuracy of the ordinal scores was measured as the degree of coincidence between ${\tilde{s}}_{i}$ and *s*_*i*_.

### Prediction of the segregation of target traits

Based on the simulated segregation of marker genotypes, we predicted the segregation pattern of target traits in a progeny population. Specifically, we sampled the values of regression coefficients, i.e., *β*_0_ and *β*_*jl*_, from their posterior distribution via MCMC using the marker genotype and trait phenotype data of the 84 cultivars. Posterior averages of the MCMC samples were used as estimates of the regression coefficients. The segregation patterns of target traits were calculated based on the marker genotypes of 1,000 progenies simulated for each cross: we calculated predicted breeding values for each progeny using Equation (1) and the estimated regression coefficients.

To estimate the degree of uncertainty of the prediction, we calculated the posterior distribution of the proportion of progenies that fulfill the selection criteria. The distribution of the proportion was calculated via the MCMC sampling of regression coefficients. More specifically, regression coefficients sampled at the *n*th MCMC cycle, i.e., *β*_0[n]_ and *β*_*jl*[n],_ were recorded through the MCMC sampling. Based on the regression coefficients, we calculated a MCMC sample of the breeding value of the simulated progeny *i* as

$${\tilde{g}}_{i\left[n\right]}=\beta_{0\left[n\right]}+\sum_{j=1}^{J}\sum_{l=1}^{L}(x_{\mathrm{ijl}}+x{'}_{\mathrm{ijl}})\beta_{\mathrm{jl}\left[n\right]}.$$

An MCMC sample of the expected breeding value of the parental variety *p* was calculated similarly. Then, we calculated a MCMC sample of the proportion of progenies that fulfilled the criteria. For example, in this study, an MCMC sample of the proportion of progenies having earlier harvest time and larger fruit weight than the cultivar ‘Kosui’ was calculated as

$$\frac{\sum_{i}^{N}I\left({\hat{g}}_{1i\left[n\right]}<{\tilde{g}}_{1p\left[n\right]}\right)I\left({\hat{g}}_{2i\left[n\right]}>{\tilde{g}}_{2p\left[n\right]}\right)}{S},..$$

where *S* is the number of simulated progenies, ${\hat{g}}_{1i\left[n\right]}$ and ${\hat{g}}_{2i\left[n\right]}$ respectively stand for the predicted breeding value of the progeny *i* in harvest time and fruit weight, and where ${\tilde{g}}_{1p\left[n\right]}$ and ${\tilde{g}}_{2p\left[n\right]}$ respectively denote the expected breeding values of the cultivar ‘Kosui’ in harvest time and fruit weight. *I*(**·**) is an indicator function having value 1 if the conditional equation is true and the value 0 otherwise. The posterior distribution of the proportion of progenies was obtained by aggregating the MCMC samples of the proportion calculated as described above.

### Validation based on data of a real breeding population

To validate the proposed method, we applied the method to predict the segregation pattern of harvest time and fruit weight in a real breeding population. The breeding population was an F_1_ progeny population derived from ‘Akiakari’ × ‘Taihaku’. The phenotypic values of harvest time and fruit weight of this population were measured in 2010 and 2011, as described earlier. We calculated the posterior distributions of probabilities obtaining progenies fulfilling the criteria for harvest time (i.e., earlier harvest time than ‘Kosui’) and fruit weight (i.e., larger fruit weight than ‘Kosui’), and compared the posterior distributions with the realized proportions of progenies fulfilling the criteria in 2010 and 2011.

### Validation based on simulated data

We performed simulations based on the real marker data of the 84 Japanese pear cultivars. We simulated 30 QTL at 30 of the 333 markers. These 30 markers were selected randomly. Genotypes of the QTL were assumed to be the same as the selected markers. Additive effects were assigned to the alleles of the QTL so that the distribution of additive genetic variances contributed by QTL followed a geometric series as defined in Eq. 10 in [53]. No dominance or epistatic effect was simulated. The effective number of QTL [53] was set as 10. The additive effects of all the QTL were summed to calculate the genotypic values of the 84 cultivars. Environmental variation was then added so that trait heritability (i.e., the fraction of the sum of additive genetic variances of all the QTL to the phenotypic variance) was set to 0.75. No genotype by environment interaction was simulated. Actual trait heritability varied because of linkage disequilibrium (LD) between the sampled QTL. Therefore, we sampled QTL repeatedly until the total heritability fell within the range of 0.70 to 0.80. We randomly paired the 84 cultivars to groups of two, and generated 42 F_1_ populations of 1,000 individuals. Using the marker and simulated phenotypic data of the 84 cultivars, we built a prediction model. In the simulations, we assumed that phenotypic variations were recorded directly as continuous data, and applied the Equation (1) directly to the data. Using the proposed method, we predicted the proportion of progenies that fulfill the selection criterion, having a larger breeding value than the average of the 84 cultivars. Finally, we compared the true and predicted proportions of progenies fulfilling the criteria. The accuracy was measured as Pearson’s product moment correlation coefficients between the true and predicted proportion. We repeated the simulation 100 times.

## Competing interests

The authors declare that they have no competing interests.

## Authors' contributions

HI conducted the study and wrote the manuscript. TH supervised the statistical aspect of the study and helped the simulation study. ST and TY collected the molecular data and helped to draft the manuscript. NT and TS collected the phenotypic data and helped to draft the manuscript. All authors read and approved the final manuscript.
